# Awareness among nurses concerning the human papilloma virus in the selected clinics in Vhembe district of Limpopo Province, South Africa

**DOI:** 10.3389/fpubh.2025.1627425

**Published:** 2025-09-19

**Authors:** Matodzi Pertunia Mushasha, Lebitsi Maud Modiba

**Affiliations:** ^1^Department of Health Studies, College of Human Sciences, University of South Africa, Pretoria, South Africa; ^2^Department of Public Health Pharmacy and Management, School of Pharmacy, Sefako Makgatho Health Science University, Pretoria, South Africa

**Keywords:** human papillomavirus, human papillomavirus screening, and vaccination, HPV awareness, HPV vaccination awareness, HPV screening awareness

## Abstract

**Background:**

HPV infection is a common sexually transmitted infection that can cause cervical cancer if left untreated. According to the World Health Organisation, in 2022, cervical cancer, resulting from HPV infection, ranked as the fourth most common cause of death for women globally. There were approximately 660,000 new cases and approximately 350,000 deaths attributed to this disease. Globally, HPV is responsible for approximately 90% of cervical cancers diagnosed in women, which is a leading cause of cancer-related morbidity and mortality. In addition to cervical cancer, HPV is also linked to a significant proportion of other anogenital cancers (such as vaginal, vulvar, anal cancers) and some oropharyngeal cancers. However, HPV is not associated with breast cancer, which is another common cancer among women. This study aimed to determine the awareness of HPV infection screening and vaccination among nurses in Vhembe district of Limpopo province, South Africa.

**Methods:**

This study utilised a quantitative approach and a cross-sectional descriptive design.115 registered nurses from 15 randomly selected clinics in the Vhembe district. Data collection was carried out through self-administered questionnaires, and analysis was performed using the Statistical Package for the Social Sciences (SPSS) version 26. Descriptive statistics were used to summarise the collected data, with outcomes presented through frequency charts and tables.

**Results:**

Of the 115 respondents, 78.3% of the respondents reported that they had never received any formal training on HPV infection screening procedures, such as how to perform the screening or sample collection for HPV infection testing. The study results also revealed that the majority, 60% of the respondents, were unaware that HPV infection does not always manifest its signs and symptoms. Of 115 respondents, the majority, 79.1%, were unaware that HPV infections do not always require treatment. The majority of the 86.1% of respondents believed that HPV infection does not cause cervical cancer. On the contrary, 13.9% of the respondents believed that HPV infection can indeed cause cancer of the cervix.

**Conclusion:**

The study concluded that nurses have a low level of awareness of HPV infection. Furthermore, it is recommended that HPV infection screening short training programmes are designed for newly qualified nurses and experienced nurses to improve their knowledge. Limited understanding of HPV infection among nurses may contribute to patients receiving insufficient information about HPV infection.

## 1 Introduction

The human papillomavirus (HPV) infection is a leading cause of sexually transmitted infections. HPV is primarily transmitted through sexual contact, making it the most common sexually transmitted infection globally. While other non-sexual transmission routes have been suggested in literature, including vertical transmission and contact with contaminated surfaces, sexual activity remains the predominant route of transmission for HPV (1). While some women may experience HPV infections that resolve spontaneously without treatment, others may develop complications such as cervical cancer, which can be fatal if not treated promptly, particularly if diagnosed at an advanced stage (2). Human papillomavirus (HPV) comprises over 200 types, causing genital warts, as well as various cancers, including those affecting the throat, cervix, and anus (3). Cervical cancer prevention primarily depends on screening and vaccination. HPV screening is used to detect the presence of high-risk HPV types that are associated with cervical cancer. However, it does not measure the severity or stage of the disease. Instead, it helps to identify individuals who may require further diagnostic procedures such as colposcopy or biopsy (4, 42). The World Health Organisation and the South African Department of Health advocate for routine cervical screening, including cytology and HPV DNA testing, depending on availability and infrastructure. These methods aim to detect abnormal cervical changes early, thus improving the prognosis through timely intervention (4, 5). In rural areas like Vhembe district in Limpopo province, uptake remains low due to limited awareness, cultural barriers, and poor access to healthcare (6). The Centres for Disease Control and Prevention emphasise HPV infection screening significance in sexually transmitted infections, with differing levels of severity among HPV types. Although some infections may resolve spontaneously, others can lead to complications such as cervical cancer, underscoring the importance of HPV infection screening services for women’s health due to daily exposure to its transmission routes (7). It was stated that HPV infection signs and symptoms that persist without disappearing on their own, HPV becomes necessary to determine the type of the virus, whether is high risk HPV type or not. As HPV infections remain to be a global contributor to cervical cancer with significant regional variation. HPV is responsible for approximately 90% of cervical cancers diagnosed in women, which is a leading cause of cancer-related morbidity and mortality. In addition to cervical cancer, HPV is also linked to a significant proportion of other anogenital cancers (such as vaginal, vulvar, anal cancers) and some oropharyngeal cancers. However, HPV is not associated with breast cancer, which is another common cancer among women (3). A study was done in Asia on knowledge about human papillomavirus and cervical cancer prevention among nurses, it was revealed that the majority 39.5% of respondents were uncertain whether HPV vaccination has major side effects or not and this finding suggest insufficient awareness regarding HPV vaccination (8). A supporting study was also conducted in Israel among nurses, majority 60.5% of the respondents, were not aware of the different body regions that can be affected by HPV and this finding concludes that training of nurses on HPV infection is very crucial (9). A cross-sectional study of knowledge, attitudes, barriers and practices of cervical cancer screening among nurses in selected hospitals in the Eastern Cape Province, South Africa, it was stated that only 16.0% nurses showed awareness that HPV vaccines is highly recommended to girls less than 16 years and 84.0% of nurses believed that HPV vaccine is highly recommended to girls above 16 years (10). This lack of knowledge was also marked on the study done at Nelson Mandela Bay clinics, where the majority of nurses, 63.64%, stated that condoms are not protective measures against HPV infection transmission (11). A continent-wide cross-sectional survey among healthcare providers from 23 African countries (including South Africa) found that only 9.9% of providers answered key HPV vaccine knowledge questions correctly, and most reported a lack of training in counselling or recommending HPV vaccination (12). Since the national rollout of South Africa’s HPV vaccination programme in 2014, efforts have largely focused on school-based delivery to Grade 4 girls, achieving high coverage but with limited emphasis on structured training of nurses, the key frontline implementers of vaccination and screening (13, 14). A 2022 narrative review highlighted that despite improving uptake rates, formal capacity-building efforts targeting healthcare workers, especially nurses, remain under-documented, which could compromise providers’ ability to promote HPV prevention services effectively (14). A South African regional audit conducted in 2023 revealed widespread poor knowledge and limited engagement in screening activities among nurses in the Eastern Cape, with negative attitudes towards HPV vaccination also reported, underscoring urgent needs for system-level interventions (10).

In South Africa, provincial data revealed that Limpopo province is one of the provinces with the highest HPV infection prevalence rate at 59% following Gauteng with 64% and Eastern cape with 63% (15). A study revealed that over 84,000 women screened for HPV infection in Limpopo 19.6% were found to have abnormal cells in the cervix. Furthermore, women who were HIV positive were found to have high prevalence of HPV infection at 31.8% compared to HIV negative women at 9.2% (16). Similar results were reported from the study conducted in Eastern cape among women, where high risk HPV infection prevalence was reported at 28.5% with high rates among HIV. positive women at 40.6 and 21.4% among HIV negative women. The study also points out several behavioural risk factors contributing to this high prevalence, including having more than three lifetime sexual partners, unprotected sexual activity and unusual virginal discharge (17). These results highlight the need for targeted interventions in provinces like Limpopo, where challenges such as inadequate access to healthcare, high HIV rates, and insufficient cervical cancer screening contribute to an increased risk of HPV-related health issues. In South Africa, a national school-based HPV vaccination programme was introduced in 2014, targeting girls aged 9–14 before sexual debut. Two vaccines are used: the bivalent vaccine (effective against high-risk HPV types 16 and 18 linked to cervical cancer) and the quadrivalent vaccine (effective against types 6 and 11, which cause genital warts). These vaccines also offer protection against other cancer-related HPV types. The programme aims to reduce HPV prevalence and cancer risk, with the WHO recommending two doses administered 6 months apart (18). In 2017, the South African Department of Health introduced primary HPV DNA testing as part of the national cervical cancer screening strategy, aiming to improve early detection of high-risk HPV types in women (19). Nurses are often the first point of contact for patients in public healthcare facilities and are responsible for implementing these services. However, if nurses lack adequate knowledge and training on HPV, its transmission, and prevention strategies, including the importance of HPV testing the success of these programmes may be compromised. Therefore, this study aimed to determine the awareness about HPV infection screening and vaccination among nurses in Vhembe district of Limpopo Province, South Africa. The population for this study were nurses with expertise in health promotion, patient education and delivery of preventative health care services. Nurses’ knowledge and attitude play a role in influencing the public’s seeking health behaviour and trust in medical intervention. Addressing gaps in awareness is important as insufficient awareness among nurses can undermine the National Department of Health’s effort in the reduction of HPV infections and cervical cancer rate. The findings of this study can therefore inform public health policy and contribute to the design of targeted educational interventions and training for healthcare professionals.

## 2 Materials and methods

### 2.1 Study design

This study utilised a quantitative research method, involving the counting, measuring, and statistical analysis of data. Employing a cross-sectional descriptive survey design allowed the collection of extensive data from a single location simultaneously (20).

### 2.2 Study setting

This study was conducted specifically within Thulamela Municipality, one of the four local municipalities in the Vhembe District of Limpopo Province, South Africa. Thulamela covers a largely rural area, with a population of approximately 618,462 people living in over 140,000 households (21). The municipality includes both peri-urban and deeply rural communities, many of which experience challenges in accessing preventive healthcare services such as HPV infection screening and vaccination. Thulamela was selected due to its high population density, the presence of multiple public healthcare facilities, and its representativeness of rural health access challenges common in northern Limpopo. The study focused solely on health facilities within this municipality. This study included nurses working in public healthcare clinics under Thulamela Municipality who were willing to sign the consent form, and nurses not working under this Municipality and unwilling to sign the consent form were excluded. In this study, the population consisted of registered nurses (RNs), registered auxiliary nurses (RANs), and registered enrolled nurses (RENs) who worked in the 15 selected clinics under Thulamela municipality, Vhembe district. The total population of nurses was 115. The population consisted of all registered nurses with ≥2 years working experience and working in selected clinics in the Vhembe district.

### 2.3 Sampling and sampling procedure

The study population was relatively small; therefore, a census approach was employed whereby all available respondents were included. Specifically, RNs, RANs, and RENs within the selected clinics were targeted. For the sampling of clinics, probability sampling was utilised to ensure that each clinic within the Thulamela municipality had an equal chance of inclusion. A simple random sampling technique was implemented to select 15 clinics from a total of 49. Each clinic was assigned a unique identifier, and the identifiers were mixed thoroughly. The researcher then manually selected 15 identifiers at random to form the sample. This process ensured that every clinic had an equal chance of being included.

### 2.4 Data collection instrument

The researcher used a questionnaire as the tool to collect data. All questions were closed-ended and formulated in English. The questionnaire was developed from another study that assessed knowledge, attitude, and awareness of HPV and modified to accommodate all the objectives of this study (6, 43). The questionnaire consisted of 3 sections: Section A covered socio-demographic data of respondents, Section B included general questions about HPV infection awareness, and Section C addressed awareness regarding HPV infection triage and test of cure. In Sections B and C, respondents had to indicate whether a statement was true or false.

### 2.5 Validity and reliability

Validity ensures the research’s integrity, ensuring that a reliable tool gauges what it is intended to assess and accurately assesses genuine distinctions among variables, excluding any consistent errors (22). In the study, where the questionnaire was used, face validity was confirmed through a process involving a two-group peer review survey. This process ensured that the scope was fully covered and that the questions were appropriately structured. Reliability refers to the degree to which the data collection tool, when used to collect information, can produce consistent results when applied on two separate occasions (22). The dependability of the questionnaire used in this study was ensured through the stability (test–retest) method. The test–retest method was used in this study, where identical questionnaires were administered to the same respondents with a 5-day interval between administrations on two occasions. This helped to determine whether the instrument would produce the same results as it had on the first occasion. Respondents received the questionnaires on two different occasions to avoid memorisation of the questionnaire. A correlation coefficient near 1 (one) signifies strong reliability, while a coefficient near zero indicates weak reliability (22).

### 2.6 Data collection procedure

The researcher collected data with the research assistant, who visited the selected clinics in the Vhembe district of Thulamela municipality after receiving ethical clearance. The respondents received details of the study, that is, how the study would be conducted, any risks and advantages of the research to the respondents, which were clarified with the respondents before beginning with data collection. Respondents, who expressed their willingness to participate in the study, received a consent form. By signing the form, they agreed to participate in the research. Data collection was done at the selected clinics in the Vhembe District under Thulamela municipality. The nurses who were to participate in the study received questionnaires to complete during lunch time or during their free time, which took 25 to 30 min to complete. The researcher distributed and collected the questionnaires on the same day to minimise non-responses and to prevent respondents from not returning questionnaires.

### 2.7 Data analysis

Data analysis is defined as exploring and organising the collected data to have meaningful results (23). The collected data was analysed using SPSS software version 26 for both cleaning and analysis purposes. Data cleaning was performed to remove any errors within the dataset. During the data analysis phase, the information was organised, categorised, and condensed to derive meaningful insights. Descriptive statistics were used to summarise the collected data, and the outcomes were presented through frequency charts and tables.

### 2.8 Ethical considerations

Ethical clearance was obtained from the University of South Africa (reference number: 63279401-CREC-CHS-201), and further permission to conduct the study in selected Thulamela clinics was granted by the Limpopo Department of Health (reference number: LP-202201-021) and the Vhembe District office (reference number: S5/4/2/3). Respondents were provided with an information letter outlining the study’s purpose, aims, potential benefits, and risks. The researcher verbally explained this information, and informed consent was obtained in writing from those who agreed to participate. Furthermore, in this study, the respondents who participated were 18 years and older, and therefore could give their consent on their own, as stated in the Constitution of the Republic of South Africa. Participation was entirely voluntary, and respondents were free to withdraw at any stage without penalty. Confidentiality and anonymity were ensured by using unique respondent codes, e.g., 001, instead of personal identifiers. All data were securely stored under lock and key to prevent unauthorised access. The study design and questionnaire were carefully developed to avoid causing any emotional, physical, or social harm to the respondents.

## 3 Results

The data were collected from 115 respondents. Descriptive analysis was conducted to acquire the percentages and frequency distribution of socio-demographic data, involving aspects such as occupation, age, gender, HPV infection screening, and HPV infection workshop/training. As well as inferential statistics analyses. In this study, the researcher returned to the selected clinics and gave the questionnaire back to the respondents on two different occasions to determine whether the results would be the same. In this research study, Cronbach’s alpha coefficient was 0.638, which was deemed acceptable as the correlation coefficient approached 1 (one). A summary of this socio-demographic data was compiled and presented in Table 1 and Supplementary Table S1 with supplementary analysis without male data and Table 2 represent general awareness regarding HPV infection, with Supplementary Table S2 representing supplementary analysis without male data. Only variables that demonstrated a statistically significant positive correlation with HPV infection screening and vaccination practices were included in the correlation analysis presented in Table 3 and non-significant variables were disregarded. Only variables with statistically significant relationships were included i non-significant variables were omitted.

**Table 1:** Socio-demographic data of respondents (*n* = 115).

Demographic characteristics	Frequency	Percentage
Gender		
Females	106	92.2%
Males	9	7.8%
Age		
20–24 years	3	2.6%
25–35 years	17	14.8%
36–45 years	29	25.2%
46–55 years	53	46.1%
56–66 years	12	10.4%
67–75 years	1	0.9%
Type of record		
RN	76	66.1%
REN	19	16.5%
RAN	20	17.4%

**Table 2:** HPV infection general awareness examination (*n* = 115).

HPV infection general awareness examination	Percentage	Frequency
HPV is associated with the development of cervical cancer		
True	99	86.1%
False	16	13.9%
Engaging in multiple sexual relationships increases the likelihood of contracting HPV infection		
True	102	88.7%
False	13	11.3%
HPV infection can be transmitted through sexual intercourse		
True	76	66.1%
False	39	33.9%
An individual might have HPV infection for an extended period without being aware of it		
True	101	87.8%
False	14	12.2%
HPV infection does not always manifest obvious signs or symptoms		
True	46	40.0%
False	69	60.0%
HPV infections are not very common		
True	59	51.3%
False	56	48.7%
There are many types of HPV		
True	86	74.8%
False	29	25.2%
Men cannot get HPV infection		
True	48	41.7%
False	67	58.3%
Consistent condom use can reduce the risk of contracting HPV infection		
True	75	65.2%
False	40	34.8%
HPV infection can spread from one individual to another through direct skin-to-skin contact in the genital area		
True	64	55.7%
False	51	44.3%
HPV is responsible for the development of genital warts		
True	79	68.7%
False	36	31.3%
Antibiotics can be used to treat HPV		
True	60	47.8%
False	55	52.2%
HIV/AIDS is caused by HPV		
True	41	35.7%
False	74	64.3%
Most people who are sexually active will likely encounter HPV infection at some stage in their lifetime.		
True	79	68.7%
False	36	31.3%
Engaging in sexual activity at a young age increases the likelihood of contracting HPV infection		
True	98	85.2%
False	17	14.8%
Typically, HPV infection does not require any treatment		
True	24	20.9%
False	91	79.1%
HPV infection screening		
Have taken HPV infection screening	66	57.4%
Have never taken HPV infection screening	49	42.6%
Previously trained for HPV infection screening		
Never	90	78.3%
Seven-twelve months ago	7	6.1%
Thirteen-twenty-four months ago	3	2.6%
≥2 years	15	13.0%

**Table 3:** Correlation coefficients (r) and significance (p) between independent variables and the dependent variable related to HPV infection and HPV vaccine knowledge.

Independent variables	Dependent variables			
	HPV infection does	HPV infections are	Man	Engaging in sexual intercourse at young
	not always	not very	cannot	age increases the risk of contracting HPV infections
	manifest	common	get HPV infections	(r,p)
	signs and	(r,p)	(r,p)	
	symptoms			
	(r,p)			
Age	_	_	R = –0201	R = –0.218
			*p* = 0.031	*p* = 0.020
Gender	R = –0.225 *p* = 0.016	_	_	_
Profession	_	R = –0.196 *p* = 0.035	_	_

### 3.1 Socio-demographic data of the respondents

As indicated in Table 1, the research findings showed a significant gender imbalance, with 92.2% of respondents being female nurses and only 7.8% male nurses. The respondents’ ages ranged from 20 to 75 years, with the majority, 46.1%, between 46 and 55 years of age, followed by 25.2% respondents between the ages of 36–45 years, and 14.8% ranging from 25 to 35 years. A small proportion of 10.4% were between 56 and 66 years. A minimal number of 2.6% were between 20 and 24 years old, and only 0.9% were in the 67–75 age bracket. And the majority were RNs at 66.1%, while 17.4% were RANs. The socio-demographic data of the respondents are presented in Table 1.

### 3.2 General awareness of HPV infection

As shown in Table 2, the study outcomes indicated that the majority, 86.1% of the respondents, believed that HPV did not lead to cervical cancer. On the contrary, 13.9% of the respondents believed that HPV can indeed cause cancer of the cervix. The findings of this study demonstrated that the majority of 88.7% of nurses were aware that being sexually involved with multiple partners increased susceptibility to HPV infection, and a single sexual encounter is sufficient to transmit HPV infection. On the contrary, only 11.3% of nurses were unaware that being sexually involved with multiple partners increased susceptibility to HPV infection. The findings of the study indicated that the majority of the survey respondents, 66.1% knew that HPV infection can be passed from one person to another through unprotected of sexual activity, including anal, oral, and vaginal sex. Furthermore, HPV infection transmission can also occur even after a single encounter. 33.9% of the respondents lacked knowledge of how HPV infection is transmitted.

This research demonstrated that 87.8% knew that an individual can have HPV and remain unaware for several years. On the contrary, only 12.2% of the respondents showed a lack of understanding of this characteristic of HPV. Respondents with a lack of knowledge on HPV infection also lack awareness of the information regarding HPV. The study outcomes revealed that 60.0% of respondents were unaware that HPV infection may not always present with visible symptoms. In contrast, 40.0% correctly identified that HPV infection may remain asymptomatic, indicating a moderate level of awareness regarding the virus’s manifestation. A supplementary analysis excluding male respondents was conducted to explore gender-specific awareness patterns. For example, in response to the statement “HPV infection does not always manifest obvious signs or symptoms,” the number of false responses was higher when all participants were included. However, when only female data were considered, the true responses increased, indicating greater awareness among respondents. Further details are provided in Supplementary Table S2.

A statistically significant correlation was found between gender and knowledge of HPV infection symptom presentation, with a Pearson’s correlation coefficient of −0.225 and a *p*-value of 0.0016, suggesting that knowledge levels varied by gender. The study outcomes showed that 51.3% respondents believed that HPV infections are uncommon, while 48.7% of the respondents believed that HPV infections are not uncommon. A significant relationship was noted between the profession of the respondents and HPV infection rarity, indicated by a p-value of 0.0035 and Pearson’s coefficient of −0.196. The findings of this study indicated that a significant majority of 74.8% of the respondents were aware of the reality of multiple types of HPV. In contrast, 25.2% of the respondents indicated that they did not understand the various types of HPV. The investigation shows that 41.7% of nurses showed a low level of awareness that men are also at risk of developing HPV infection, while 58.3% of the respondents lacked knowledge that men could be infected by HPV infection. A significant negative correlation was also found between age and the gender at risk of HPV infection, with a *p*-value of 0.031 and Pearson’s correlation of 0.201. The study concluded that most of 65.2% of the respondents were aware that using condoms can decrease the risk of contracting HPV infection. On the contrary, 34.8% of the respondents showed a low level of awareness that the use of condoms can lower the likelihood of contracting HPV infections. The findings of this research also demonstrated that 55.1% knew about the transmission of HPV infection via direct skin-to-skin contact in the genital area. On the contrary, 44.3% of the respondents showed a lack of awareness that HPV infection could be transmitted in this manner. The results showed that 68.7% of nurses had a high level of awareness that HPV can cause the development of warts in the genital area. Additionally, 31.3% showed a low level of awareness that HPV may cause the development of genital warts.

The outcomes of this research indicated that the majority of 52.2% of the respondents believed that antibiotics can be used to treat some of the symptoms caused by HPV infection, while 47.8% respondents were aware that HPV infection symptoms cannot be treated by antibiotics. The collected data indicated that 35.7% of nurses show a lack of awareness that HPV infection is not responsible for causing Human Immunodeficiency Syndrome/Acquired Immunodeficiency Syndrome (HIV/AIDS). In addition, 64.3% of the nurses show a high level of awareness that HPV infection is not responsible for HIV/AIDS. In this research study, 68.7% of nurses showed a high level of awareness that sexually active people are at high risk of contracting HPV infection in their lives, and this virus can be contracted through only one episode of unprotected sexual intercourse. Furthermore, 31.3% of nurses showed a low level of awareness that sexually active people have a high probability of contracting HPV infection in their lives.

The study findings revealed that a significant majority of 85.2% of nurses were conscious that engaging in sexual intercourse at a young age increases the likelihood of acquiring HPV infection. Moreover, the exposure to HPV infection may happen after a single sexual experience, while a small proportion of only 14.8% were unaware of this connection. A significant negative relationship was observed between age and knowledge about the risk of engaging in sexual intercourse at a younger age, with a Pearson correlation coefficient of −0.218 and a *p*-value of 0.020. The research highlights that most respondents, 79.1%, were unaware that HPV infections do not always require treatment. Although 0.9% of the respondents showed a sufficient level of awareness that HPV-related infections can disappear without treatment. The research indicated that 57% of the respondents underwent HPV infection screening, while 42.6% of the respondents never underwent HPV infection screening. The collected data indicated that most of the respondents, 78.3%, had never received any training on HPV infection screening. A smaller proportion of 13.0% of the respondents had received HPV screening training more than 2 years ago, while 6.1% had received HPV infection screening training in the last six to 12 months. Furthermore, 2.6% of nurses obtained HPV screening training 12–24 months ago. The HPV infection general awareness examination is illustrated in Table 3.

## 4 Discussion

According to the results of this study, nurses showed a low level of awareness and understanding regarding the human papillomavirus. Limited HPV infection awareness among nurses can impact patients/women negatively. The more nurses know about HPV infections, the more patients/women are also aware of HPV infections and able to take preventive measures against HPV infections.

The findings revealed that the respondents in the study were predominantly females, with males’ representation being minimal. The high level of female nurses may be due to the belief and attitude of male nurses that they will be called ‘sisters’ as their female counterparts are called in the profession. However, they can be referred to as ‘professional nurse Mushasha” instead of “sister Mushasha.” In a study conducted in urban India, it was found that of 318 respondents, 71 were men and 247 were women (24). Another study in South Africa also found mostly female healthcare workers. This study looked at the knowledge of HPV in Nelson Mandela Bay clinics. Just like in this current study, there were many more women 106 than males 9 in that South African study (25). The study participants represented a wide age range, with the majority falling between the ages of 46 and 55. Younger and older age groups were less represented. The results of this research study concur with those found in another study conducted by Ma et al. (26), which found that the average age of the respondents was 21 years old.

A greater proportion of RNs participated in the study compared to RENs and RANs, suggesting that the perspectives captured are more representative of the RN category. The results of this study are in line with the findings of the study carried out at the Nelson Mandela Bay Clinic in the Eastern Cape, South Africa. The study results revealed that among the 110 nurses who participated in the study, 79 were RNs. However, the RENs numbered more than the RANs (25). The study revealed that just over half of the respondents had undergone HPV infection screening, indicating moderate uptake. However, a substantial portion had never been screened, underscoring the need to improve screening coverage among the target population. A study carried out in China on HPV infection screening and vaccination among university students found that 98.4% of respondents had done HPV infection screening, and 1.6% of students had never done HPV infection screening (26). These findings validated the findings of this study. The findings revealed a significant gap in HPV infection screening training among respondents, with the majority indicating they had never received such training. Only a small number reported having been trained within the past 2 years, highlighting the need for ongoing professional development in this area. The findings of a study, which examined the perspectives of primary health care professionals (HCPs) on the introduction of cancer screening services in a tribal region of Maharashtra, India, confirmed the outcomes of this study, in that most of the respondents never received training in cancer (27).

The findings revealed a widespread misconception among respondents, with most not recognising the link between HPV and cervical cancer. This highlights a critical gap in knowledge that may hinder effective prevention and early detection efforts. In this study, the results contradicted the outcomes of the study carried out in Western China, which reported that 53.4% of the 1,109 respondents were aware that HPV could cause cancer of the cervix, and 46.6% were unaware of which diseases were caused by HPV (28). The findings of this study demonstrated that the majority of the nurses knew that being sexually involved with multiple partners increased susceptibility to HPV infection. These findings support those found in a study conducted by Cocchio et al. (29), it was revealed that of 9,188 respondents, the majority, 83.3%, were aware and had high levels of knowledge that having multiple sexual partners increased the risk of contracting HPV infection.

The study showed that while the majority of respondents understood that HPV infection is transmitted through sexual contact, including anal, oral, and vaginal sex, a notable proportion remained unaware of these transmission routes. This highlights ongoing gaps in knowledge that need to be addressed through targeted education, unlike a previous study in Limpopo Province, where most people, 85.1%, did not know how HPV infection spreads. In that study, some mistakenly thought that kissing, 3.2% or unprotected sex, 2.1%, could transmit HPV infection. Furthermore, 9.60% of the respondents stated that sexual intercourse is the main mode of HPV infection transmission (30). The study revealed that most participants were aware that HPV infection can remain undetected for years, suggesting a fair level of understanding regarding the virus’s asymptomatic nature. However, a small portion still lacked this awareness, indicating a need for continued education. The results align with a study conducted in the United Kingdom; the study found a similar awareness among Health Care Professionals (HCPs), 634 of 643 knew HPV infection can go undetected for a long time. Just like in this current study, only a small number of 9 were unaware of this fact (31). The study found that 40.0% of the respondents knew that HPV infection may not always show symptoms. On the contrary, 60.0% were unaware of this. There is a significant negative correlation between gender and knowledge regarding the manifestation of HPV infection signs and symptoms, with a Pearson correlation coefficient of −0.225 and a *p*-value of 0.016. This further suggests that men may ignore obvious signs and symptoms of HPV infection and never consult to receive treatment. Moreover, women who are mostly seen going to clinics to consult, which means that any signs and symptoms of HPV infection are likely to consult to receive treatment. The findings are similar to another study in Al Qunfudhah, both studies found that most respondents were unaware that HPV infection can be symptomless. In this current study, 60% were unaware, and the Al Qunfudhah study found 65% unaware (32). Participants were divided in their beliefs about how common HPV infections are, reflecting uncertainty or gaps in knowledge about their widespread nature. Lack of knowledge is seen to lower nursing categories (assistance and auxiliary nurses). Another significant relationship was found between profession and HPV infection rarity, indicated by a *p*-value of 0.035 and a Pearson’s correlation coefficient of −0.196. This suggests that nurses of a higher category, such as registered nurses, may possess sufficient awareness about HPV infection compared to enrolled nurses. This may influence registered nurses to protect themselves from being infected by HPV infection compared to enrolled nurses due to the level of education they have. The results of this study contradicted those found by Rahman et al. (33), who stated that the majority of the respondents were aware that HPV infections are common, while 32.3% were unaware that HPV infections are not rare.

The study found that most respondents were aware of the existence of multiple HPV types. Notably, awareness was higher among nurses with over 5 years of experience and those who had received HPV infection screening training, suggesting that both experience and targeted education play a crucial role in improving HPV-related knowledge. The conclusions of this research contrast with those of the earlier study on Moroccan women’s knowledge, perception, and willingness to receive the HPV vaccine. The study found that few women were aware of HPV and its different types. 131 respondents had better awareness, while 759 respondents had no awareness about HPV and its types (34). The findings revealed that a considerable number of nurses lacked awareness that men are also at risk of contracting HPV. This knowledge gap highlights the need for inclusive HPV infection education that addresses both male and female vulnerability to the virus. A significant negative correlation was found between age and knowledge regarding the gender at risk of HPV infection, with a *p*-value of 0.031 and a Pearson correlation coefficient of −0.201. Older individuals with a high level of knowledge are likely to understand that both men and women are at risk of contracting HPV infection and are likely to take preventative measures against HPV.

There was a lack of knowledge about HPV infections related to the awareness of HPV, its infections, and prevention. Seyfettinoglu & Hark (35) conducted a study on physician attitudes on HPV vaccination of their son; the results contradict the findings of this study by indicating that 100% of respondents were aware that men can also be diagnosed with HPV infection. The study revealed that while most respondents were aware that condom use can reduce the risk of contracting HPV infection, a significant portion still lacked this knowledge. This suggests the need for more comprehensive education on preventive measures against HPV infection transmission. Women who think they are at low risk of contracting HPV infection are not likely to use preventative measures that can prevent them from contracting HPV infection. The conclusions support those of a previous study, which reported that among 670 respondents, 408 were informed that the protective role in the use of condoms in sexual activity reduced the risk of being infected by HPV. Furthermore, 262 respondents were unaware that the use of condoms can protect against HPV infection or not (36).

The findings also revealed that while just over half of the respondents were aware that HPV infection can be transmitted through direct skin-to-skin contact in the genital area, a substantial proportion lacked this knowledge. This gap highlights the need for clearer communication about the modes of HPV infection transmission in healthcare training. Women’s beliefs and attitudes also play a role in how they look at certain diseases and how they can affect them. The results of this study concurred with those of a study conducted by Tian et al. (37), which indicated that a substantial proportion of respondents 296 demonstrated awareness of HPV infection transmission through skin-to-skin contact, while 140 respondents expressed uncertainty about the transmission mode. The results revealed generally low awareness among nurses regarding the link between HPV and the development of genital warts. Only a small proportion demonstrated a high level of understanding, indicating a need for improved training on the clinical manifestations of HPV. Insufficient knowledge of HPV infections can result in women being ignorant of the severity of the disease and how it can affect their lives and health (36), which corroborates the results of the study, which indicated that of the 670 respondents in the study, 592 were aware and aware that genital warts are caused by HPV. Few respondents, 78, had no understanding of the complications of HPV infections. Furthermore, it was indicated that the lack of knowledge was linked to the fact that the undergraduate courses were not related to health sciences.

The findings revealed mixed understanding among respondents regarding the treatment of HPV-related infections. While some believed that antibiotics could be used, nearly half lacked awareness or held misconceptions about appropriate treatment approaches. This highlights the need for clearer education on the management of viral infections caused by HPV. A woman who is aware of HPV and its infection, or has a history of having HPV infection, or has a family member who suffers from HPV infection, may identify these as cues to action, which can lead to understanding that antibiotics cannot be used to treat HPV infection. Sypie & Zielonka (38) supported the results of this study, indicating that among 302 respondents, 216 were aware that HPV infection signs and symptoms cannot be treated with anti-infectives. 86 respondents were unaware that anti-infectives cannot be used to treat HPV infection. This study found that parents with higher HPV infection knowledge, more education, and those living in large cities were more likely to know about HPV vaccination. These parents were also more likely to support the vaccine, view it as safe and effective, and be willing to vaccinate their children. The study identified a notable knowledge gap among nurses regarding the relationship between HPV and HIV/AIDS. While many nurses demonstrated correct understanding, a significant number of participants mistakenly believed that HPV causes HIV/AIDS. This highlights the need for clearer differentiation between the two viruses in educational programmes. This lack of awareness about HPV infection can cause women to have misconceptions about the virus, its causes, effects, and prevention methods. These misconceptions might negatively impact their attitudes towards HPV infection and screening programmes (6), which corroborates the findings of this research study by stating that 211 of the 227 respondents showed a high level of awareness that HPV cannot cause HIV/AIDS, and 29 respondents showed a low level of awareness that HPV is not responsible for causing HIV/AIDS. The level of HPV knowledge concerning HPV infection among HCPs in New Zealand was observed to be inadequate and incomplete, showing a lack of training. In this study, 68.7% of nurses showed a high level of awareness that sexually active people are at high risk of contracting HPV infection in their lives. The study identified a significant knowledge gap about HPV among nurses. This lack of awareness about HPV infection can lead women to have misconceptions about the virus, including their risk of infection. Women who underestimate their risk of HPV infection may be less likely to take preventive measures such as condoms, potentially increasing their exposure to the virus. Tanaka et al. (39) conducted a study, which contradicted the findings of this study. Of 152 pregnant adolescents, 48 respondents were aware that sexually active people are at high risk of contracting HPV and may develop HPV infection in their lifetime. It was indicated that girls with a low level of knowledge and education are engaged in risky sexual behaviour.

The findings demonstrated that most nurses were aware of the link between early sexual activity and an increased risk of acquiring HPV infection. However, a small proportion remained unaware, suggesting the need to reinforce this aspect in sexual health education. Women’s belief that there is no possibility of being infected with HPV may lead them to engage in early sexual intercourse. A significant negative relationship was observed between age and knowledge about the risk of engaging in sexual intercourse at a younger age, with a Pearson correlation coefficient of −0.218 and a *p*-value of 0.020. As people grow, they understand the risks of contracting HPV due to lived experiences, and they are more likely to take preventative measures against HPV infection, such as using condoms and having one sexual partner. Chanprasertpinyo & Rerkswattavorn (40) conducted a study, and the results differ from the results of this study, in that of 521 respondents, 273 were aware that engaging in sexual intercourse increased the risk of contracting HPV infection, while 248 were unaware that engaging in sexual activity at a young age increased the probability of acquiring HPV. The study highlighted that peer pressure among adolescents contributes to early participation in sexual activity, increasing the risk of HPV infection transmission. Furthermore, it revealed that the majority of respondents, 79.1%, did not know that HPV infections do not always require treatment. The study found that majority of the respondents, did not know that HPV infections can clear up on their own. This suggests a lack of education from healthcare providers, which could impact women’s understanding of HPV and the importance of prevention. George et al. (41) confirm the finding of this study by indicating that only 146 were aware of HPV infection and its vaccines. Furthermore, 12 respondents were aware that HPV does not always require treatment, and 134 respondents were unaware that HPV infections does not always require treatment. It was indicated that a lack of awareness and knowledge was identified among the respondents, the majority of whom lacked awareness that they were at the age where they should know about HPV, HPV vaccination, and prevention to limit HPV infections.

## 5 Conclusion

The study findings underscore a concerning lack of awareness among registered nurses about HPV screening and vaccination. This knowledge gap highlights a potential shortfall in healthcare provision, as nurses play a crucial role in patient education and preventive care. Consequently, it is imperative to implement HPV infection training programmes aimed at newly qualified nurses and experienced physicians to enhance their understanding and proficiency in managing HPV-related issues. By improving nurses’ knowledge through targeted education initiatives, healthcare facilities can ensure that patients receive complete and accurate information on HPV, its risks, and preventive measures. Failure to address this limited understanding of HPV infections among nurses may not only compromises patient care but also perpetuates inadequate awareness and understanding of HPV infections within the broader community. Thus, investing in ongoing education and training for nurses is essential to improve public health outcomes and combat the spread of HPV infections.

## Data Availability

The raw data supporting the conclusions of this article will be made available by the authors, without undue reservation.
