# Supplementary material for: Awareness among nurses concerning the human papilloma virus in the selected clinics in Vhembe district of Limpopo Province, South Africa
Source: Front Public Health. 2025 Sep 19;13:1627425. doi: 10.3389/fpubh.2025.1627425 (PMC12491301; doi:10.3389/fpubh.2025.1627425)
Supplement: Supplementary file 1 [file Table_1.pdf]

Supplementary table

Table S1: Socio-demographic data (Supplementary analysis without males' data) (n=106)

Demographic data	Frequency	Percentages
**Gender**		
Females	106	100
**Age**		
20-24 years	2	1.9%
25-35 years	15	14.2%
36-45 years	28	26.4%
46-55 years	49	46.2%
56-66 years	11	10.4%
67-75 years	1	0.9%
**Type of record**		
RN	73	68.9%
REN	29	27.4%
RAN	4	3.8%
