# Supplementary material for: Awareness among nurses concerning the human papilloma virus in the selected clinics in Vhembe district of Limpopo Province, South Africa
Source: Front Public Health. 2025 Sep 19;13:1627425. doi: 10.3389/fpubh.2025.1627425 (PMC12491301; doi:10.3389/fpubh.2025.1627425)
Supplement: Supplementary file 2 [file Table_2.pdf]

Supplementary table

Table S2: HPV general awareness examination (Supplementary analysis without male data) (n=106)

HPV general awareness	Frequency	Percentage
**HPV is associated with the development of cervical cancer**		
True	93	87.7%
false	13	12.3%
**Engaging in multiple sexual relationships increases the likelihood of contracting HPV**		
True	93	87.7%
False	13	12.3%
**HPV can be transmitted through sexual intercourse**		
True	69	65.1%
False	37	34.9%
**An individual might have HPV for an extended period without being aware of it**		
True	94	88.7%
False	12	11.3%
**HPV does not always manifest obvious signs or symptoms**		
True	66	62.3%
False	40	37.7%
**HPV is not very common**		
True	55	51.9%
False	51	48.1%
**There are many types of HPV**		
True	79	74.5%
False	27	25.5%
**Men cannot get HPV**		
True	46	43.4%
False	60	56.6%
**Consistent condom use can lower the risk of contracting HPV**		
True	71	67.0%
False	35	33.0%

**HPV can spread from one individual to another through direct skin-to-skin contact in the genital area**		
True<br>False	57<br>49	53.8%<br>46.2%
**HPV is responsible for the development of genital warts**		
True<br>False	71<br>35	67.0%<br>33.0%
**Antibiotics cannot treat HPV**		
True<br>False	52<br>54	49.0%<br>50.9%
**HIV/AIDS is caused by HPV**		
**The majority of individuals who are sexually active will likely encounter HPV at some stage in their lifetime**		
True<br>False	72<br>34	67%<br>17%
**Engaging in sexual activity at a young age raises the likelihood of contracting HPV**		
True<br>False	89<br>17	84%<br>16%
**Typically, HPV does not require any treatment**		
True<br>False	22<br>84	20.8%<br>79.2%
**HPV screening**		
Have taken HPV screening<br>Have never taken HPV screening	62<br>44	58.5%<br>41.5%
**Previously trained for HPV screening**		
Never<br>Seven- twelve months ago Thirteen-twenty-four months ago<br>≥2 years	82<br>8<br>3<br>13	77.4%<br>7.5%<br>2.8%<br>12.3%
